# Affective Shifts Outside Work: Effects on Task Performance, Emotional Exhaustion, and Counterproductive Work Behavior

**DOI:** 10.3389/fpsyg.2021.640144

**Published:** 2021-05-31

**Authors:** Xingyu Qu, Xiang Yao, Qishuo Liu

**Affiliations:** School of Psychological and Cognitive Sciences, Beijing Key Laboratory of Behavior and Mental Health, Peking University, Beijing, China

**Keywords:** positive affect, negative affect, affective shift, task performance, emotional exhaustion

## Abstract

Affective shifts have been linked to work attitudes and behaviors recently, but previous researches only focused on affective shift during work, with little attention to affective shifts outside work. Conservation of resources and personality system interaction theories are used to design a 2-week daily dairy study. Participants report how affective shifts outside work affect their subsequent-day task performance, emotional exhaustion, and CWB. As expected, findings indicate that shifts in affect outside work meaningfully impact job performance and work attitudes. That is, when both positive and negative affect upshift outside work, employees perform their tasks better but also experience increased emotional exhaustion. Practical implications and limitations are discussed.

## Introduction

*Affect* is defined as “a phase of neurobiological activity that is experienced as motivational and informational and that influences thought and action” (Izard, 2009, p. 3). *Positive affect* (PA) indicates positive feelings such as passion, relaxation, and pleasure; *negative affect* (NA) indicates negative feelings such as anger, guilt, and fear (Watson et al., 1988). Affect is known to influence work behaviors (Ashkanasy and Dorris, 2017), but researchers have always measured affect levels at specific timepoints. However, affect will change or shift over time, which leads to affective shift. Affective shift can be interpreted as the fluctuations of PA or NA within individual during a specific time period (Gross, 1998; Yang et al., 2016), which indicates that we should investigate how changing affect influences work performance.

Three particularly relevant published papers have explored how affective shifts occurring during work generate positive work outcomes. For example, Bledow et al. (2011) found that NA experienced in the morning positively relates to work engagement in the afternoon, but only if work engagement generates high PA. In a followup study, Bledow et al. (2013) demonstrated that decreased NA coupled with increased PA generates high creativity. Yang et al. (2016) found that upshifted PA accompanied by downshifted NA predicted better organizational citizenship behavior (OCB), and upshifts in both PA and NA predicted better task performance. In all those studies, the authors calculated affective shift by measuring PA and NA twice daily and used the fluctuations between the two time points to represent affective shift (Bledow et al., 2013; Yang et al., 2016). They also focused on affective shifts during worktime only. Thus, we still lack understating toward affective shifts, about how affective shifts outside work influence work behaviors. After-work affective shift differs from after-work recovery. Affective shift indicates fluctuating emotions (Yang et al., 2016), while after-work *recovery* captures revitalization though leisure activities, with positive effects on next-day work performance (Sonnentag, 2003).

Though after-work recovery has been widely tested, we still lack researches investigating the effect of after-work affective shifts on work behaviors. More tests are needed regarding affective shift outside work. First, though affective shifts during work has been tested, there lacks researches concerning affective shifts outside work. PA and NA can spillover from work to home and vice versa (Kinnunen et al., 2014; Wei et al., 2018; Kopperud et al., 2020), so that affect experienced outside work could influence subsequent work performance (Judge and Ilies, 2004). We need to study how affective shifts outside work influence subsequent work behavior and performance.

In addition, Yang et al. (2016) overlooked emotional exhaustion as an important affect-related outcome variable. Indeed, affect state fluctuations can be emotionally exhausting (Ashkanasy and Dorris, 2017). Thus, affect-related research frameworks should always consider emotional exhaustion as an underlying influence on other outcome variables such as job satisfaction (Grandey and Gabriel, 2015).

Furthermore, Yang et al. (2016) investigated affective shifts only during work for influences on OCB, without considering an opposite performance outcome: counterproductive work behavior (CWB). From a definitional and empirical perspective, harmful CWBs and beneficial OCBs (Dalal, 2005) should both be considered when examining impacts of affective shifts during and after work. Task performance, OCB, and CWB are considered separate performance domains (Rotundo and Sackett, 2002), but to understand how outside-work affective shift impacts job performance, we include CWB and thus expand the limited focus on task performance and OCB (Yang et al., 2016).

**FIGURE 1:**
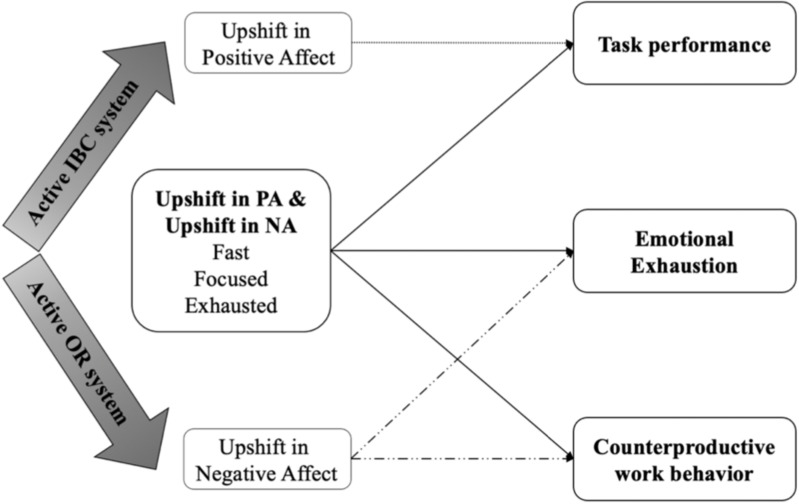
Hypothese for the current study. Dotted line indicates Hypothese 1. Top dashed line indicates Hypothese 2. Bottom dashed line indicates Hypothese 3. Top solid line indicates Hypothese 4. Bottom solid line indicates Hypotheses 5 and 6.

We conducted this research to fill those gaps in understandings of affective shift. Drawing from conservation of resources (COR) theory (Hobfoll, 1989, 2002) and personality system interaction (PSI) theory (Kuhl, 2000), we examine our hypotheses regarding impacts of affective shift outside work on task performance, emotional exhaustion, and CWB. By offering a more balanced perspective regarding affective shift during and outside work, we make important theoretical contributions to the literature on affect shift, work behavior, and well-being. First, we extend the work of Yang et al. (2016) by providing empirical evidence showing how outside-work affective shift impacts job performance. We extend PSI theory by applying it to non-work situations and using resources’ perspective to view activated subsystems from PSI theory. Second, our research also contributes to the literature on emotional exhaustion and CWB, which are both essential indicators of job performance in affect-related research (Rotundo and Sackett, 2002; Ashkanasy and Dorris, 2017). In simultaneously studying the effects of after-work PA and NA shifts, our research provides a finer grained picture of the powerful ways in which even after-work affective shift can influence these important work behaviors. Third, we use daily dairy study design and collect variables separated in timepoints. This allows us to establish temporal precedence and provide us stronger support for hypothesized causal relationships in correlational research. To better understand and visualize our hypotheses, we refer to the Figure 1 of Yang et al. (2016) to draw our hypotheses. Figure 1 lists our hypotheses.

## Theory and Hypotheses

### Affective Shift Outside Work

Conservation of resources theory (COR theory) basically assumes that people try to gain resources, to protect the resources they have, and to regain resources they have lost. Potential resource losses cause anxiety and stress, which then influence behaviors (Hobfoll, 1989, 2002). The relationship with stress has been widely identified in both work and non-work areas, such as emotional labor (Grandey and Gabriel, 2015), leadership (Usman et al., 2020), and recovery (Kinnunen et al., 2011).

The theory implies that work life potentially spills over to home life and vice-versa (Wei et al., 2018; Kopperud et al., 2020). Empirical studies have indicated that affect shift outside work influences next-day work attitudes and behaviors. For example, employees who engage in after-work recreation show better work performance and satisfaction, but employees who fail to participate in non-work activities show poorer work-related outcomes (Kinnunen et al., 2014). Employees who participate in after-work sporting or entertainment activities show decreased NA because they recover mental resources consumed during work (Grandey and Gabriel, 2015). They also gain PA that then spills over to their work life (Edwards and Rothbard, 2000).

Personality system interaction theory explains that self-regulation of affect has dynamic effects on cognition, motivation, and personality (Kuhl, 2000). The theory is usually applied to working contexts (e.g., Yang et al., 2016), but studies are increasingly showing that working and non-working contexts have closely related crossover and spillover effects (Williams and Alliger, 1994; Lin et al., 2017; Yang et al., 2018; Cluley and Hecht, 2019). For example, employees who showed helping behavior at work were more likely to feel PA that then caused them to be more supportive of their spouses (Lin et al., 2017). When rating family satisfaction and quality of family life, the most positive ratings came from spouses of employees who indicated having increased self-esteem because they had socially supportive servant leaders in the workplace (Yang et al., 2018). In contrast, distress experienced during family activities and family intrusions into work spilled over to perceptions of family interference with work (Williams and Alliger, 1994). Stress associated with families, spouses (Williams and Alliger, 1994), and children (Cluley and Hecht, 2019) were shown to increase work-family conflict and damage work outcomes (Wei et al., 2018; Kopperud et al., 2020). In addition, after-work recovery was shown to influence work-time performance or psychological variables such as work engagement, proactive behavior, and fatigue (Sonnentag, 2003; Kinnunen et al., 2011). In summary, studies suggest that work and non-work are inseperable and that PSI theory applies in both contexts.

Our hypotheses are based on COR theory (Hobfoll, 1989, 2002) and PSI theory (Kuhl, 2000). PSI theory identifies four automatic subsystems that motivate individual behaviors according to positive and NA (Kuhl, 2000). That is, individuals use the (1) intuitive behavior control system (IBC) to integrate wide information for rapid and intuitive problem solving; (2) objective recognition system (OR) to judge whether observations match previous representations or present new concepts; (3) intention memory system (IM) to make comprehensive plans and predict outcomes; (4) extension memory system (EM) to integrate stored representations of internal and external contexts with personal experiences and values. IBC and OR systems are lower-level systems, while IM and EM systems are higher-level systems. The subsystems have mutual influences (Kuhl, 2000).

**FIGURE 2:**
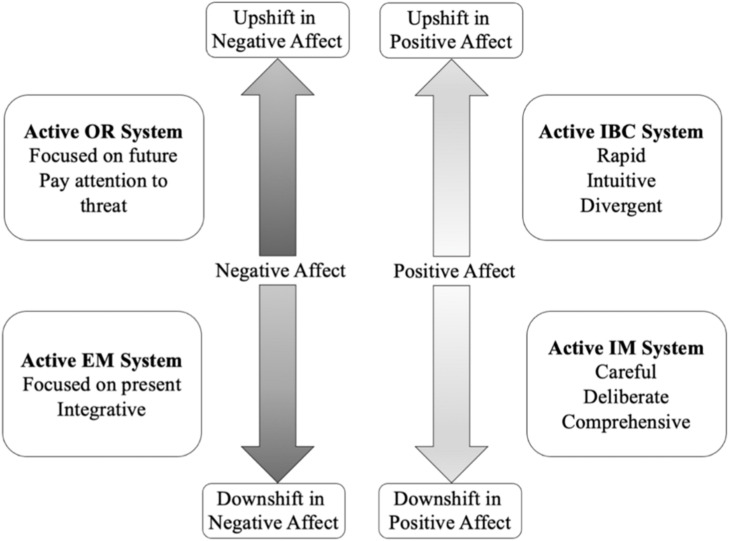
Activation subsystem of affective shift in positive and negative affect from PSI theory.

Personality system interaction theory emphasizes that affective shift has specific impacts on each subsystem, ultimately enhancing or decreasing motivational regulation and leading to optimal or suboptimal behavior. Figure 2 shows how affective shift relates to the four subsystems. Specifically, upshifts in PA activate the IBC system, so that the individual is motivated to use intuitive, rapid, divergent thinking. Downshifts in PA activate the IM system, so that the individual uses comprehensive thought, careful plans, and deliberate actions. Upshifts in NA activate the OR system, so that the invidual focuses on specific details before assuming threats and need for actions. Downshifts in NA activate the EM system, so that the individual focuses on the present and integrates information from different sources.

#### Affective Shift and Task Performance

We use both conservations of resources theory (Hobfoll, 1989, 2002) and PSI theory (Kuhl, 2000) to build our hypotheses. Task performance, an important indicator of job performance, refers to how well employees perform their roles and contribute to organizational development (Yang et al., 2016). COR theory (Hobfoll, 1989, 2002) explains that individuals who have high PA perform better because they are better able to acquire and accumulate resources, broaden their mindsets, adopt explorative behaviors, make remote associations (Hobfoll et al., 2018), be cognitively flexible, be explorative, master more skills, and devise more creative solutions (Conway et al., 2013). In addition, resource-rich individuals have greater resilience for recovering from negative stimuli such as conflict, frustration, or even the strain of positive activities (Hobfoll et al., 2018). For example, resilience is essential for service industry employees who must deal with frequent customer complaints and maltreatment. In addition, PSI theory explains that upshifted PA improves task performance by activating IBC systems that involve rapid and divergent learning, thinking, and problem solving; along with open-minded, explorative, and flexible mindsets (Lucas et al., 2014). Thus, we hypothesize that PA upshifts outside work replenish resources that are used to enhance job performance (Fredrickson, 1998):

*Hypothesis 1*: Shifts in PA outside work are positively correlated with task performance the next-day, such that upshifts in PA outside work will promote task performance.

#### Affective Shift and Emotional Exhaustion

Emotional exhaustion, the core dimension of job burnout, is a response to stress (Grandey, 2003) in which energy is depleted and emotional resources dissipate (Cordes and Dougherty, 1993; Khan et al., 2019). Recall that COR theory identifies the need to acquire and preserve resources (Hobfoll, 1989, 2002). And the loss of resources in one domain causes a spiral of rapidly depleted resources in other domains (Usman et al., 2020). Thus, the resources consumed by NA upshifts would accelerate the loss of resources in other domains. NA upshifts are represented as emotionally exhausting as potential losses in evoking feelings of threat, reducing well-being, inducing dysfunctional thinking (Halbesleben et al., 2014; Hobfoll et al., 2018), and inhibiting abilities to cope with work demands.

Personality system interaction theory explains that NA upshifts activate the OR system linked with increased alertness, decreased goal desirability, increased disengagement, and attention to threats such as impending deadlines (Rothbard and Wilk, 2011). NA upshifts will cause individuals to ruminate about upcoming threats rather than take action against them, leading to anxiety, tension, and resource depletion. Thus, we hypothesize:

*Hypothesis 2*: Shifts in NA outside work are positively correlated with emotional exhaustion the next-day, such that upshifts in NA outside work will increase emotional exhaustion.

#### Affective Shift and Counterproductive Work Behavior

Counterproductive work behavior indicates the dark side of job performance dimensions (Rotundo and Sackett, 2002). That is, CWBs are intentional actions that damage organizational functions or member relationships (Spector and Fox, 2002). CWBs include actions such as theft, sabotage, overt anger, and passive-aggressive poor performance (Meier and Spector, 2013). CWB-I indicates interpersonally oriented CWB, such as gossip; CWB-O indicates organizationally oriented CWB such as taking prolonged breaks during work time (Robinson and Bennett, 1995; Bennett and Robinson, 2000).

Negative affect is strongly related to CWB. That is, employees with high NA are prone to CWB (Dalal, 2005), but to clarify the relationship between NA shift and CWB (Spector and Fox, 2002), we argue that upshifted NA leads to more CWB by consuming resources. To reiterate, COR theory explains that individuals strive to protect, acquire, and accumulate resources, but make stronger efforts to prevent losses. NA upshifts are consuming resources and accelerating the loss of resources in other domains (Usman et al., 2020). Depleted resources result in a loss of self-control. The increased potential for CWB can be exhilirating and self-reinforcing when it serves as retaliation against organizations or colleagues (Spector and Fox, 2002; Meier and Spector, 2013). In addition, PSI theory states that NA upshifts activate OR system, leading employees ruminate about upcoming threats rather than take action against them. In working situation, the rumination without action to solve upcoming threats may be considered as CWB. Thus, when after-work NA increases, employees will react to the loss of resources and decreased self-control by increasing next-day CWB, which leads to our third hypothesis:

*Hypothesis 3*: Shift in NA outside work is positively correlated with CWB the next-day, such that upshift in NA outside work will increase CWB.

### Interplay of Affective Shifts

Personality system interaction theory (Kuhl, 2000) takes a new perspective by showing that affect undergoes changes rather than remaining constant, with differing interplaying patterns in the four subsystems (for specifics, see Yang et al., 2016). Yang et al. (2016) defined these four patterns as PA upshift and NA downshift (pattern A), PA and NA upshifts (pattern B), PA downshift and NA upshift (pattern C), PA and NA downshifts (pattern D), respectively. For our purposes, however, we focus on the pattern B defined by Yang et al. (2016), which is the most common after-work PA and NA upshift patterns.

Outside work activities are essential for relaxing, recovering, and replenishing resources (Sonnentag, 2003) and thus increasing PA (Hobfoll et al., 2018). However, even during after-work hours, employees cannot psychologically detach or “switch off” because they are often assailed with work-related internet and instant messages. As they ruminate about work, they are likely to have upshifted NA (Casper and Sonnentag, 2019).

Positive affect upshift activates the IBC system governing intuitive, integrative information processing, encouraging the generation of solutions and actions for resolving problems (Kuhl, 2000). Indeed, PA upshifts have been shown to increase motivation, goal commitment (Ilies and Judge, 2005), and goal striving efforts (Locke and Latham, 2006). However, PA upshift also indicates self-satisfaction about having met goals, which may decrease effort (Carver and Scheier, 1990). At this point, NA upshift becomes important for activating OR systems that draw attention to possible threats (Kuhl, 2000; Rothbard and Wilk, 2011), such as deadlines. Thus, PA upshift without NA upshift may generate exploratory but impractical behaviors, whereas NA upshift without PA upshift may evoke anxiety but not goal desirability or effort.

Thus, PA and NA upshifts occurring simultaneously activate both IBC and OR systems. Activated IBC systems generate open-minded proposals for solutions and rapid actions. Activated OR systems focus the attention on threats, rapid action, and quick error correction. When employees have demanding/stressful work days, they often stay cognitively active and continue processing work-related information after work hours. They need time to unwind and to cease ruminating about work-related issues (Casper and Sonnentag, 2019). Thus, non-work time can be utilized to process work-related information. In addition, research of affective shift during work has shown that PA and NA upshifts during work could significantly predict better subsequent-day task performance (Yang et al., 2016).

We propose that NA upshifts will determine whether PA upshifts increase task performance. That is, when the IBC and OR systems are activated, employees will be focused on solutions and details and will perform their best, leading to our fourth hypothesis:

*Hypothesis 4*: Change in NA outside work moderates the relationship between change in PA outside work and task performance next-day. Specifically, an upshift in PA will positive correlated to task performance when there is a corresponding upshift in NA.

We have explained that simultaneous upshifts in PA and NA activate IBC and OR systems so that employees act rapidly and efficiently, promptly correct errors, and provide quality work. Beyond the positive outcomes, however, simultaneous PA and NA upshifts may have some negative outcomes in that rapid, high quality work is demanding and depletes resources, which then accelerates the loss of resources in other domains (Usman et al., 2020). Without adequate recovery, employees are eventually emotionally exhausted and no longer able to perform well (Hobfoll et al., 2018). Lacking resources, they will lack self-control (Vötter and Schnell, 2019). Such resource depletion is one of the best predictors of CWB (Dalal, 2005). Thus, we propose:

*Hypothesis 5*: Change in PA outside work moderates the relationship between change in NA outside work and emotional exhaustion next-day. Specifically, upshifted NA will be more positively related to emotional exhaustion when there is a upshift in PA outside work.

*Hypothesis 6*: Change in PA outside work moderates the relationship between change in NA outside work and CWB the next-day. Specifically, an upshift in NA will be more positively related to CWB when there is a corresponding upshift in PA.

## Method

### Participants and Procedure

In this study, our participants were call-center employees who routinely interact with customers and inevitably experience social conflicts that will evoke negative thoughts about work during non-work times (Volmer et al., 2012). They were ideal subjects to examine whether NA upshifts combined with PA upshifts will increase task performance. That is, whether activation of the IBC and OR systems will cause employees to focus on detailed solutions and perform their best.

We examined how after-work activities influence next-day attitudes at work (Judge and Ilies, 2004). In many professions, telework has blurred boundaries between activities that occur during and after work (Standen et al., 1999). Consequently, our study of call-center employees was appropriate because their work has clear boundaries: they work in specific offices, keep regular work hours, and use fixed-line telephones. We focused on affective shifts occurring between afternoons after work and mornings before work the next-day.

Serving as a liaison, the human resource manager of a telecommunications company in southern China independently and randomly selected 80 fulltime call-center employees. We then sent email invitations to introduce the project, explain the procedure, promise confidentiality, and offer rewards for participation.

Sixty-eight employees volunteered to participate, but only 64 provided usable data. First, they completed a pre-test capturing Big-5 personality traits, PA/NA traits, and demographics such as age and gender. During the two following weeks, they completed online surveys twice daily: once in the morning before starting work at approximately 8 a.m., and once at the end of the work day before leaving the office at approximately 5 p.m. We chose a 2-week period based on recommendations to record for 2 weeks to ensure “a stable and generalizable estimate of social life” (Reis and Wheeler, 1991, p. 287). The research team transmitted online reminder messages before each scheduled survey. Following previous research (Bledow et al., 2013; Yang et al., 2016), we surveyed participants at 8 a.m. to measure the beginning-of-work timepoint and again at 5 p.m to measure the end-of-work timepoint. Participants completed both morning and afternoon surveys for an average of 10.72 days, generating 686 sets of matched morning and evening observations. To test our hypotheses, we used 622 matched sets of prior-afternoon, following-morning, and following-afternoon observations, 607 of which were complete and usable. Among the participants, 92% were women, averaging 29.78 years-old (SD = 3.49) and 15.21 years of education (SD = 1.64). Approximately 10% had high school diplomas; 46% had associate’s degrees; 28% had bachelor’s degrees; and 16% had graduate degrees.

### Measures

All instructions and scale items were written in Chinese through a translation–back translation approach (Brislin, 1983). In the pre-test, participants reported their Big-5 personality traits, PA and NA traits, and demographics. During the morning surveys, they reported PA and NA states and the quality of sleep they attained the preceding night. During the afternoon surveys, they reported PA and NA states, emotional exhaustion, task performance, and CWBs.

#### PA/NA

We used the positive and negative affect scale (PANAS) (Watson et al., 1988) to measure PA and NA. The pre-test included ten items each for measuring general PA and NA. To increase participation and reduce survey fatigue in the diary studies, we selected three items for each dimension: *delighted*, *excited*, and *active* for PA; *angry*, *guilty*, and *upset* for NA. Researchers have found that using PANAS in daily surveys over 10 working days is suitable for ensuring compliance (Bledow et al., 2011, 2013; Yang et al., 2016). Participants reported their current feelings on a five-point scale from 1 (*not at all*) to 5 (*extremely*). For morning affect state, Cronbach’s alpha averaged across 10 work days was 0.97 for PA and 0.77 for NA. For afternoon affect state, average alpha was 0.94 for PA, and 0.79 for NA. For affect trait, Cronbach’s alpha was 0.87 for PA and 0.84 for NA.

For morning affect state, the multilevel alphas for PA were 0.92 at the within-person level and 0.99 at the between-person level; for NA they were 0.73 at the within-person level and 0.80 at the between-person level (Geldhof et al., 2014). For afternoon affect state, the multilevel alphas for PA were 0.91 at the within-person level and 0.97 at the between-person level; for NA they were 0.72 at the within-person level and 0.86 at the between-person level.

#### Emotional Exhaustion

Emotional exhaustion was measured using a nine-item scale developed by Maslach and Jackson (1984). Participants rated how extensively they agreed with item descriptions such as “The work I did today frustrated me.” Responses were measured on a seven-point scale from 1 (*not at all*) to 7 (*extremely*). Cronbach’s alpha across 10 days was 0.94. The multilevel alphas for emotional exhaustion were 0.92 at the within-person level and 0.96 at the between-person level.

#### Task Performance

Task performance was measured using a five-item scale developed by Janssen and Van Yperen (2004). Participants rated how extensively they agreed with items such as “Today I carried out all the responsibilities required by work,” on a seven-point scale from 1 (*not at all*) to 7 (*extremely*). Cronbach’s alpha across 10 days was 0.78. The multilevel alphas for task performance were 0.65 at the within-person level and 0.87 at the between-person level.

#### Counterproductive Work Behavior (CWB)

Counterproductive work behavior was measured using a 14-item scale with two dimensions, eight items for CWB-I and six items for CWB-O, developed by Dalal et al. (2009). Participants rated their agreement with items for CWB-I such as “Today I occasionally spoke ill of my supervisor/colleagues behind their backs” and with items for CWB-O such as “Sometimes I conduct sabotage during work,” on a seven-point scale from 1 (*not at all*) to 7 (*extremely*). Cronbach’s alpha across 10 days was 0.95.

Cronbach’s alpha for CWBI across 10 days was 0.973; for CWBO it was 0.949. The multilevel alphas for CWBI were 0.94 at the within-person level and 0.99 at the between-person level. As for CWBO, multilevel alphas were 0.90 at the within-person level and 0.97 at the between-person level.

#### Control Variables

We considered age, gender, years of education, PA/NA traits, and sleep quality as control variables. Sleep quality was measured with a single-item, “How was your sleep last night?” on a five-point scale from 1 (*very poor*) to 5 (*very good*).

### Construct Validity

To ensure that the variables were distinct constructs, we ran multilevel confirmatory factor analyses in Mplus 8.11 (Muthèn and Muthèn, 1998-2012). Results for a seven-factor model encompassing daily PA/NA, emotional exhaustion, performance, CWB and total PA/NA (PANAS) were, χ^2^(899) = 8186.9, *p* < 0.001, CFI = 0.76, RMSEA = 0.12, within-level SRMR = 0.10, between-level SRMR = 0.13. All factor loadings were significant. If we combine daily NA with emotional exhaustion, the six-factor model results were, χ^2^(903) = 9297.6, *p* < 0.001, CFI = 0.72, RMSEA = 0.12, within-level SRMR = 0.14, between-level SRMR = 0.13. Although the results are a poor fit for both models, the former seven-factor model fit better than the plausible alternative six-factor model combining NA and emotional exhaustion into one factor.

### Analytic Strategies

To model the relations among within-individual affective shift, emotional exhaustion, task performance, and CWB, and to control for the effects of between-individual demographics and trait affect, we used hierarchical linear modeling (HLM) (Bryk and Raudenbush, 1992), which allows variables to be analyzed at multiple levels in a series of regression equations. Our first level of analysis included the daily measures of state affect (PA and NA), emotional exhaustion, task performance, and CWBs. The second level of analysis included the measure of PA/NA trait and demographic variables. Thus, level-1 variables were nested within level-2 variables. All level-1 predictors were person mean-centered. All level-2 variables were grand-mean-centered.

To explore the main effect of affective shift, we put next-morning PA/NA into the model by controlling previous-afternoon PA/NA (Yang et al., 2016). To explore the interplay of affective shift, we calculated the standardized residual score for PA and NA first, and then group-mean-centered the residual score. Finally, we multiplied the PA residual score by the NA residual score and added the product into the model as a new variable. To calculate residual score, we regressed next-morning PA/NA on previous-afternoon PA/NA, although others (Bledow et al., 2013; Yang et al., 2016) measured PANAS twice each morning and twice at the end of work, and used the residual score change between morning and at the end of work to represent affect shift during work. Considering our focus on affective shift outside work, we also controlled for sleep quality to avoid possibilities that sleep may interfere with impacts of affect shift on work the next-day.

## Results

### Correlation Analysis

Table 1 shows means, standard deviations, and correlations. We calculated day-level correlations by HLM (Raudenbush, 2004). Previous-afternoon PA was significantly positively correlated with next-morning PA (*r* = 0.12, *p* < 0.01); next-morning PA was significantly negatively correlated with next-morning NA (*r* = −0.36, *p* < 0.01); previous-afternoon PA was significantly negatively correlated with previous-afternoon NA (*r* = −0.44, *p* < 0.01); previous-afternoon NA was significantly positively correlated with next morning NA (*r* = 0.26, *p* < 0.01); previous afternoon NA was significantly negatively correlated with next-afternoon task performance (*r* = −0.14, *p* < 0.01); previous-afternoon and next-morning NA were significantly positively correlated with next-morning CWB-I and CWB-O (*r* = 0.13, *p* < 0.01; *r* = 0.14, *p* < 0.01; *r* = 0.10, *p* < 0.05; *r* = 0.11, *p* < 0.01).

**TABLE 1:** Means, standard deviations, and correlations between focal variables.

		*M*	*SD*_1_	*SD*_2_	1	2	3	4	5	6	7	8	9	10	11	12	13
**Level-1**																	
1	Sleep quality	3.42	1.11														
2	PA (T1)	3.36	1.03		0.06												
3	NA (T1)	2.04	0.81		−0.10*	−0.44**											
4	PA (T2)	3.34	1.08		0.12**	0.12**	−0.05										
5	NA (T2)	1.99	0.79		−0.05	−0.10*	0.26**	−0.36**									
6	Task performance	5.36	1.01		0.05	0.02	−0.14**	0.07	−0.05								
7	Emotional exhaustion	4.11	1.36		−0.01	0.05	−0.05	0.01	0.07	−0.02							
8	CWB-I	2.03	1.17		−0.13**	−0.04	0.13**	−0.02	0.10*	−0.32**	0.03						
9	CWB-O	1.98	1.13		−0.13**	−0.03	0.14**	−0.07	0.11**	−0.38**	0.07	0.72**					
**Level-2**																	
10	Age	29.78		3.49	0.22	−0.02	−0.06	0.09	−0.18	−0.06	0.03	−0.01	−0.02				
11	Gender	–		–	0.05	0.22	−0.08	0.13	−0.09	−0.03	−0.11	−0.17	−0.11	−0.08			
12	Education	15.21		1.64	−0.19	−0.15	0.06	−0.13	0.11	−0.13	0.00	0.19	0.15	−0.04	−0.15		
13	PA (trait)	3.26		0.60	0.40**	0.34**	−0.11	0.37**	−0.19	0.31*	−0.18	−0.34**	−0.27*	−0.15	0.15	−0.16	
14	NA (trait)	2.75		0.64	−0.04	0.05	0.24	0.03	0.27*	0.00	0.19	0.27*	0.28*	−0.18	−0.10	0.04	−0.17

Correlations above the diagonal represent the day level (N_1_ = 607). Correlations below the diagonal represent the person level (N_2_ = 64). Day level variables were aggregated across days to calculate person-level correlations. PA/NA state labeled t1 was measured the previous afternoon; t2 was measured the next morning; Women were coded as 0; men were coded as 1.“–” indicates values not available. *p < 0.05, **p < 0.01.

Among the person-level variables, several significant correlations occurred. PA trait was significantly positively related to task performance (*r* = 0.31, *p* < 0.01) and significantly negatively related to both CWB-I and CWB-O (*r* = −0.34, *p* < 0.01; *r* = −0.27, *p* < 0.05). NA trait was significantly positively correlated with both CWB-I and CWB-O (*r* = 0.27, *p* < 0.05; *r* = 0.28, *p* < 0.05).

### Test of Hypotheses

Via HLM, we tested the main effect and interplay of affective shift on task performance/emotional exhaustion/CWB by controlling for age, gender, years of education, and PA/NA trait. First, we set the null model. Second, we put PA/NA state of t_1_ and t_2_, control variables into the model to test the main effect. Third, we put the product of affective shift into the model to test the interplay.

#### Affective Shift Outside Work and Task Performance

The null model indicated between-person variance of *T*_00_ = 0.57 (*p* < 0.001) and within-person variance of σ^2^ = 0.44: thus ICC = *T*_00_/(*T*_00_+σ^2^) = 0.57/(0.57+0.44) = 0.56. Significant between-person variance accounted for 56% of the variance in task performance.

Hypothesis 1 focused on how changes in PA outside work relate to subsequent-day task performance. Hypothesis 4 focused on how changes in PA and NA outside work interplay to affect subsequent-day task performance. Table 2 shows how affective shift affected task performance. After controlling for age, gender, years of education, and PA/NA trait, outside-work PA change was positively related to next-day task performance (β = 0.10, *p* < 0.05): when PA upshifted outside work, next-day task performance increased, supporting hypothesis 1. Change in NA outside work was not significantly related to next-day task performance (β = 0.01, *p* > 0.05). *R^2^_Level–1_* = 0.14, indicating that affective shift explained 14% of variance in task performance. Furthermore, the interaction term of the residual scores of PA and NA significantly predicted next-day task performance (β = 0.11, *p* < 0.01). *R*^2^ = 0.01, indicating that the interplay of affective shift explained 1% of task performance variance.

**TABLE 2:** Multilevel estimates for models predicting task performance.

	Step 1	Step 2	Step 3
Intercepts	5.38**	5.38**	5.40**
**Level-1**			
Sleep quality		0.07	0.07
PA (t1)		−0.03	−0.03
NA (t1)		−0.14**	−0.13*
PA (t2)		0.04	0.03
NA (t2)		−0.03	−0.04
δpa(residual) × δna(residual)			0.09*
**Level-2**			
Age		0.00	−0.00
Gender		−0.45	−0.41
Education		−0.05	−0.05
Positive affectivity		0.43**	0.45**
Negative affectivity		−0.03	−0.02
**Variance**			
σ^2^	0.44	0.34	0.33
T _00_	0.57**	0.56**	0.57**
T _11_		0.15**	0.16**
*R*^2^		0.23	
*R*^2^*_level1 interaction_*			0.03

†p < 0.1, *p < 0.05, **p < 0.01. R^2^_Level–1_ = (σ^2^ of Step 1–σ^2^ of Step 2)/σ^2^ of Step 1. R^2^_level–1 interaction_ = (σ^2^ of Step 2–σ^2^ of Step 3)/σ^2^ of Step 2.

**FIGURE 3:**
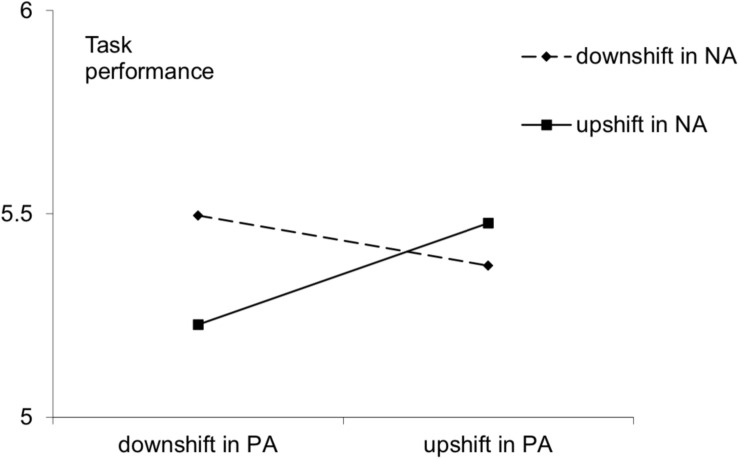
Affective shift and task performance.

Figure 3 depicts simple slopes analysis. Under low outside-work NA changes (assessed 1 *SD* below the mean of residual NA values), outside-work PA changes were non-significantly related to next-day task performance (*slope* = −0.02, *t* = −0.29, *p* > 0.05); under high outside-work NA changes (assessed 1 *SD* above the mean of residual NA values), outside-work PA changes were significantly positively related to next-day task performance (*slope* = 0.20, *t* = 3.65, *p* < 0.01). The results indicated that outside-work NA changes moderate the relationship between outside-work PA changes and task performance. When both outside-work PA and NA were upshifted, employees showed the best task performance, supporting hypothesis 4.

#### Affective Shift Outside Work and Emotional Exhaustion

Null model results indicated that between-person variance was *T*_00_ = 1.21 (*p* < 0.01), while within-person variance was σ^2^ = 0.69, thus ICC = *T*_00_/(*T*_00_+σ^2^) = 1.21/(1.21+0.69) = 0.64, showing that between-person variance caused a significant 64% of the variance in emotional exhaustion.

Hypothesis 2 focused on how outside-work NA changes relate to subsequent-day emotional exhaustion. Hypothesis 5 focused on how outside-work changes in PA and NA interplay to affect subsequent-day emotional exhaustion. Table 3 shows affective shift impacts on emotional exhaustion. After we controlled for age, gender, years of education, and PA/NA trait, outside-work NA change was not significantly related to next-day emotional exhaustion (β = 0.16, *p* > 0.05), nor was outside-work PA change related to next-day emotional exhaustion (β = 0.07, *p* > 0.05) *R^2^_Level–1_* = 0.08, indicating that the main effect of affective shift explained 8% of the variance of emotional exhaustion. Thus, hypothesis 2 was not supported. Furthermore, the interaction term of the residual scores of PA and NA significantly predicted next-day emotional exhaustion (β = 0.16, *p* < 0.05). *R^2^_level–1 interaction_* = 0.02, indicating that the interplay of affective shift explained 2% of the variance of emotional exhaustion.

**TABLE 3:** Multilevel estimates for models predicting emotional exhaustion.

	Step 1	Step 2	Step 3
Intercepts	4.11**	4.11**	4.12**
**Level-1**			
Sleep quality		−0.02	−0.02
PA (t1)		0.04	0.03
NA (t1)		−0.09	−0.07
PA (t2)		0.07	0.08
NA (t2)		0.16†	0.18†
δpa(residual) × δna(residual)			0.17*
**Level-2**			
Age		0.01	0.01
Gender		0.03	−0.05
Education		−0.01	0.01
Positive affectivity		−0.38†	−0.44*
Negative affectivity		0.28	0.24
**Variance**			
σ^2^	0.69	0.63	0.62
T _00_	1.21**	1.21**	1.18**
T _11_		0.18†	0.21†
*R^2^_Level–1_*		0.09	
*R^2^_level–1 interaction_*			0.02

†p < 0.1, *p < 0.05, **p < 0.01. R^2^_Level–1_ = (σ^2^ of Step 1–σ^2^ of Step 2)/σ^2^ of Step 1. R^2^_level–1 interaction_ = (σ^2^ of Step 2–σ^2^ of Step 3)/σ^2^ of Step 2.

**FIGURE 4:**
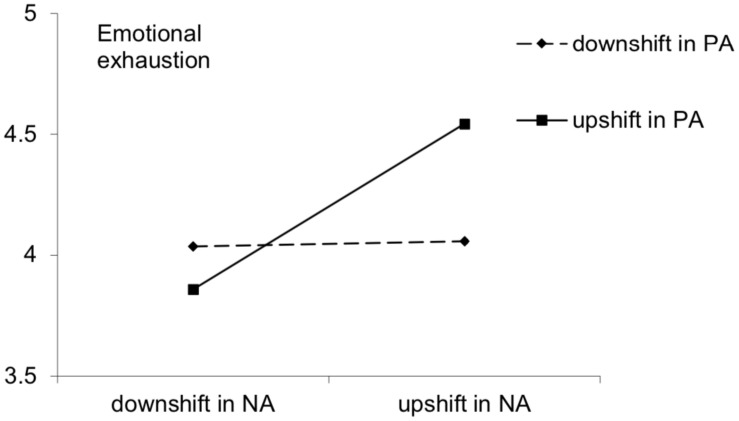
Affective shift and emotional exhaustion.

Simple slopes analysis (Figure 4) showed that under low outside-work PA changes (assessed 1 *SD* below the mean of residual PA values), outside-work NA changes were not significantly related to next-day emotional exhaustion (*slope* = 0.03, *t* = 0.20, *p* > 0.05). Under high outside-work PA changes (assessed 1 *SD* above the mean of residual PA values), outside-work NA changes were significantly positively related to next-day emotional exhaustion (*slope* = 0.34, *t* = 3.59, *p* < 0.01). The results indicated that outside-work PA changes could moderate the relationship between outside-work NA changes and emotional exhaustion. When both PA and NA were upshifted outside work, participants felt the highest emotional exhaustion, supporting hypothesis 5.

#### Affective Shift Outside Work and CWB

Null model results indicated that between-person variance was *T*_00_ = 0.84 (*p* < 0.01), while within-person variance was σ^2^ = 0.52; thus ICC = *T*_00_/(*T*_00_+σ^2^) = 0.84/(0.84+0.52) = 0.62, showing that between-person variance caused a significant 62% of CWB-I variance.

Hypothesis 3 focused on the relationship between changes in NA outside work and subsequent-day CWB. Hypothesis 6 focused on how changes in PA and NA outside work interplay to affect subsequent-day CWB. Table 4 shows how affective shift impacted CWB-I. After we controlled for age, gender, years of education, and PA/NA traits, outside-work PA change was not significantly related to next-day CWB-I (β = −0.00, *p* > 0.05), nor was outside-work NA change in relation to next-day CWB-I (β = 0.11, *p* > 0.05). *R^2^_Level–1_* = 0.14, indicating that affective shift explained 14% of CWB-I variance. The interaction term of the residual scores of PA and NA could not significantly predict next-day CWB-I (β = 0.04, *p* > 0.05).

**TABLE 4:** Multilevel estimates for models predicting CWB-I.

	Step 1	Step 2	Step 3
Intercepts	2.00**	1.99**	2.00**
**Level-1**			
Sleep quality		−0.06	−0.06
PA (t1)		0.02	0.03
NA (t1)		0.13†	0.13†
PA (t2)		−0.01	−0.01
NA (t2)		0.12†	0.12†
δpa(residual) × δna(residual)			0.01
**Level-2**			
Age		−0.00	−0.00
Gender		−0.15	−0.14
Education		0.07	0.07
Positive affectivity		−0.54**	−0.54**
Negative affectivity		0.50**	0.51**
**Variance**			
σ^2^	0.52	0.42	0.42
T _00_	0.84**	0.69**	0.70**
T _11_		0.05†	0.05
*R^2^_Level–1_*		0.19	
*R^2^_level–1interaction_*			

†p < 0.1, **p < 0.01. R^2^_Level–1_ = (σ^2^ of Step 1–σ^2^ of Step 2)/σ^2^ of Step 1. R^2^_level–1 interaction_ = (σ^2^ of Step 2–σ^2^ of Step 3)/σ^2^ of Step 2.

The null model indicated that between-person variance was *T*_00_ = 0.82 (*p* < 0.01), while within-person variance was σ^2^ = 0.45; thus ICC = *T*_00_/(*T*_00_+σ^2^) = 0.82/(0.82+0.45) = 0.65, showing that between-person variance caused a significant 65% of variance in CWB-O.

Table 5 shows how affective shift impacted CWB-O. After we controlled for age, gender, years of education, and PA/NA trait, outside-work PA change was not significantly related to next-day CWB-O (β = −0.08, *p* > 0.05), nor was outside-work NA related to next-day CWB-O (β = 0.08, *p* > 0.05); *R^2^_Level–1_* = 0.20, indicating that the main effect of affective shift explained 20% of the variance of CWB-O. The interaction term of the residual scores of PA and NA could not significantly predict next-day CWB-O (β = 0.04, *p* > 0.05). Thus, hypotheses 3 and 6 were not supported.

**TABLE 5:** Multilevel estimates for models predicting CWB-O.

	Step 1	Step 2	Step 3
Intercepts	1.96**	1.96**	1.96**
**Level-1**			
Sleep quality		−0.04	−0.03
PA (t1)		0.07	0.08*
NA (t1)		0.13†	0.13†
PA (t2)		−0.06	−0.07
NA (t2)		0.04	0.06
δpa(residual) × δna(residual)			−0.02
**Level-2**			
Age		0.00	0.00
Gender		−0.00	−0.01
Education		0.04	0.05
Positive affectivity		−0.51**	−0.49**
Negative affectivity		0.43**	0.46**
**Variance**			
σ^2^	0.45	0.33	0.32
T _00_	0.82**	0.73**	0.74**
T _11_		0.07*	0.06*
*R^2^_Level–1_*		0.27	
*R^2^_level–1interaction_*			0.03

†p < 0.1, *p < 0.05, **p < 0.01. R^2^_Level–1_ = (σ^2^ of Step 1–σ^2^ of Step 2)/σ^2^ of Step 1. R^2^_level–1 interaction_ = (σ^2^ of Step 2–σ^2^ of Step 3)/σ^2^ of Step 2.

## Discussion

In this study, we observe how shifts in PA and NA occurring outside work affect task performance, emotional exhaustion, and CWB at work the next-day. Specifically, we find that PA shift outside work is significantly correlated with next-day task performance and moderates the relationship between NA shift and emotional exhaustion. Also, PA and NA shift outside work have significant interactive effects on task performance.

Considering affective shift during worktime significantly influences subsequent-day work attitudes and work behaviors (Yang et al., 2016), and affective shift is not limited in worktime (Judge and Ilies, 2004), thus, it’s worthwhile to investigate affective shift outside work and the relationships with employees’ subsequent well-being and productivity outcomes at work. We extend affective shift to non-work time, showing how affect shifts occurring during non-work life affect work life. That is, explore after-work affective shift for its main and interplay effect on task performance, emotional exhaustion, and CWB. About the interplay effect, we apply PA and NA upshifts (pattern B) from the affective shift model as detailed in Yang et al. (2016), and an extension of that model by introducing additional outcome variables as predicted by the COR theory. Consequently, our work makes theoretical and practical contributions.

### Theoretical Implications

First, we demonstrate that affective shift outside work meaningfully impacts job performance. We provide the most recent empirical evidence upholding PSI theory arguments that PA and NA shifts could activate cognitive and behavioral subsystems that then influence work attitudes and behaviors. Although we find that PA shifts have main effects on task performance, PA and NA shifts fail to have significant main effects on emotional exhaustion or CWB. We have several explanations for those results. First, PSI theory explains that unidimensional affective shift motivates single systems only. Thus the limited effects fluctuate easily in response to external stimuli. Second, systems influence one another. Multi-systems have mutually constraining or facilitating cumulative effects. Third, our CWB results may show limited variance because participants may have avoided socially undersirable CWB.

Second, our results indicate that NA can have positive effects. When both PA and NA upshift, employees perform their best. High NA causes alertness, attention to detail, and rapid action. In our study context, call-center employees constantly solve customer problems. The company provides answers for dealing with regular questions, but detail-oriented employees provide the best service. The interplay of PA and NA shifts indicate that upshifted NA causes upshifted PA to be more positively related with task performance, while upshifted PA causes upshifted NA to be more positively related with emotional exhaustion. PSI theory (Kuhl, 2000) explains that simultaneous increases in PA and NA activate IBC and OR systems, so that employees work rapidly and well, but consumed resources lead to emotional exhaustion. The results fail to support our hypothesis regarding affective shift influences on CWB-I or CWB-O. However, PA negatively influenced CWB-O, while NA positively influenced both CWB-I and CWB-O. Thus CWB may be more susceptible to stable variables such as affective traits rather than to short-term affective shift.

Third, we explore how affective shift outside work impacts work attitudes and behaviors, we find that non-work upshift in both PA and NA can be emotionally exhausting but also improve task performance. Affect shift meaningfully impacted job performance, supporting our hypotheses that employees who enjoy upshifted PA will also acquire more resources, while those who suffer upshifted NA will consume more resources, with further negative effects on work attitudes and behaviors. Moreover, affective shift could motivate cognitive and behavioral subsystems, with influences on outcomes.

### Limitations and Future Research

Our research has four limitations that should be addressed. First, study participants self-reported all variables, risking common method bias (Griffin et al., 2007). Our longitudinal research design somewhat decreased but did not erase common method bias.

Second, we controlled for sleep quality, but it was self-reported and measured by a single item. Considering that sleepers may subconsciously shift affect by processing affect events (Walker and van der Helm, 2009; Yang et al., 2016), sleep quality may be an essential antecedent of affect (Flueckiger et al., 2016). Thus, future research could use more specific measurements such as polysomnography machines that capture actual sleep duration and quality.

Third, we did not clarify the boundary distinguishing affect during work from affect outside work. Our call-center study participants used fixed-line telephones to handle both outgoing and incoming calls. Thus, their work–non-work boundaries were easily clarified. Future research could use greater precision for controlling how prior work events influence outside-work affect.

Fourth, using PSI theory to infer hypotheses, we cannot justify the specific differences between outside-work and during-work affective shifts. Though activated subsystems are the same in upshifted PA and NA outside or during work, the antecedents may differ and patterns of resources from COR theory may vary (Hobfoll et al., 2018). For affective shift during work, there may be some work-related antecedents and resources are consumed no matter upshift in PA or NA. Whereas for affective shift outside work, there may be some non-work-related antecedents such as family activities, conflicts with spouse and so on. And resources are in a cycle of replenishment and consumption (Casper and Sonnentag, 2019). To show how outside-work and within-work affective shifts differ, future research could consider antecedents and use specific indicators to represent replenishment and consumption of resources.

### Practical Implications

Our research has practical values. Call-center employees perform emotional work in communicating directly with customers. Our findings suggest that organizations should alleviate emotional exhaustion and evoke higher performance through interventions such as emotional writing workships and regular team-building activities.

## Conclusion

In this study, we explore how affective shifts outside work impact task performance, emotional exhaustion, and CWB. We find that PA upshifts outside work are positively correlated with task performance (Hypothesis 1), that NA upshifts outside work can moderate the relationship between PA upshifts outside work and task performance (Hypothesis 4), and that PA upshifts outside work can moderate the relationship between NA upshifts outside work and emotional exhaustion (Hypothesis 5). Overall, the study indicates that affect shifts outside work have meaningful impacts on job performance and work attitudes. On a practical level, we show that human resource interventions must recognize that affect experienced after work has as much impact as affect experienced during work.

## Data Availability Statement

The raw data supporting the conclusions of this article will be made available by the authors, without undue reservation.

## Ethics Statement

The studies involving human participants were reviewed and approved by Department of Psychology. The patients/participants provided their written informed consent to participate in this study.

## Author Contributions

XQ: conceptualization, methodology, software, formal analysis, data curation, writing–original draft preparation, and writing–reviewing and editing. XY: conceptualization, resources, data curation, supervision, writing–reviewing and editing, project administration, and funding acquisition. QL: methodology, software, formal analysis, data curation, and writing–original draft preparation. All authors contributed to the article and approved the submitted version.

## Conflict of Interest

The authors declare that the research was conducted in the absence of any commercial or financial relationships that could be construed as a potential conflict of interest.
